# Caesarean delivery is associated with increased blood pressure in young adult offspring

**DOI:** 10.1038/s41598-021-89438-3

**Published:** 2021-05-13

**Authors:** Amaraporn Rerkasem, Sarah E. Maessen, Antika Wongthanee, Sakda Pruenglampoo, Ampica Mangklabruks, Patumrat Sripan, José G. B. Derraik, Kittipan Rerkasem

**Affiliations:** 1 NCD Center of Excellence, Research Institute for Health Sciences, Chiang Mai University, Chiang Mai, Thailand; 2 Liggins Institute, University of Auckland, Auckland, New Zealand; 3 Department of Internal Medicine, Faculty of Medicine, Chiang Mai University, Chiang Mai, Thailand; 4 Department of Women’s and Children’s Health, Uppsala University, Uppsala, Sweden; 5 Department of Endocrinology, Children’s Hospital, Zhejiang University School of Medicine, Hangzhou, China; 6 Department of Surgery, Faculty of Medicine, Chiang Mai University, Chiang Mai, Thailand

**Keywords:** Cardiology, Endocrinology, Medical research, Risk factors

## Abstract

We examined the associations between caesarean section (CS) delivery and cardiovascular risk factors in young adults in Thailand. Participants were 632 offspring from a birth cohort in Chiang Mai (Northern Thailand), born in 1989–1990 and assessed in 2010 at a mean age of 20.6 years, including 57 individuals (9.0%) born by CS and 575 born vaginally. Clinical assessments included anthropometry, blood pressure (BP), carotid intima-media thickness, and fasting blood glucose, insulin, and lipid profile. Young adults born by CS had systolic BP (SBP) 6.2 mmHg higher (*p* < 0.001), diastolic BP 3.2 mmHg higher (*p* = 0.029), and mean arterial pressure (MAP) 4.1 mmHg higher (*p* = 0.003) than those born vaginally. After covariate adjustments, SBP and MAP remained 4.1 mmHg (*p* = 0.006) and 2.9 mmHg (*p* = 0.021) higher, respectively, in the CS group. The prevalence of abnormal SBP (i.e., pre-hypertension or hypertension) in the CS group was 2.5 times that of those born vaginally (25.0% vs 10.3%; *p* = 0.003), with an adjusted relative risk of abnormal SBP 1.9 times higher (95% CI 1.15, 2.98; *p* = 0.011). There were no differences in anthropometry (including obesity risk) or other metabolic parameters. In this birth cohort in Thailand, CS delivery was associated with increased blood pressure in young adulthood.

## Introduction

The proportion of infants born by caesarean sections (CS) worldwide has increased markedly over recent decades, including in Thailand^1^. The reasons for this observed increase are largely unknown, but it is thought that both practitioner preference and increases in maternal request in well-resourced health systems have contributed to this trend^2^. Several studies have shown that compared to individuals born vaginally, those born by CS have an increased risk of adverse health outcomes, including overweight/obesity and hypertension in childhood, adolescence, and adulthood^3–6^. However, these findings are far from universal, with some studies showing no such associations when CS is elective^7^ or for all CS cases^8^. A meta-analysis suggested that inadequate adjustment for confounding effects and publication bias may explain the associations found in many studies, which were typically small in magnitude^9^.

While the evidence is far from conclusive, it is postulated that babies born vaginally are exposed to maternal symbiotic bacteria essential for the long-term development of a healthy gut microbiome^9,10^. One study showed that the association between maternal pre-pregnancy overweight and early offspring overweight was mediated by both birth mode and the infant’s gut microbiome, with infants of overweight mothers born by CS at the highest risk of obesity^11^. There are differences in gut microbiome between lean individuals and those with obesity in both childhood and adulthood^12^, and alterations to the gut microbiome have been shown to cause obesity in an animal model^13^. Furthermore, the gut microbiome has been associated with hypertension in animal models, and there is evidence that this may also apply to humans^14^.

In this context, we aimed to examine the associations between CS and long-term cardiometabolic outcomes in a cohort of young adults in Thailand. To our knowledge, such associations have never been previously examined in this country.

## Methods

**Figure 1:**
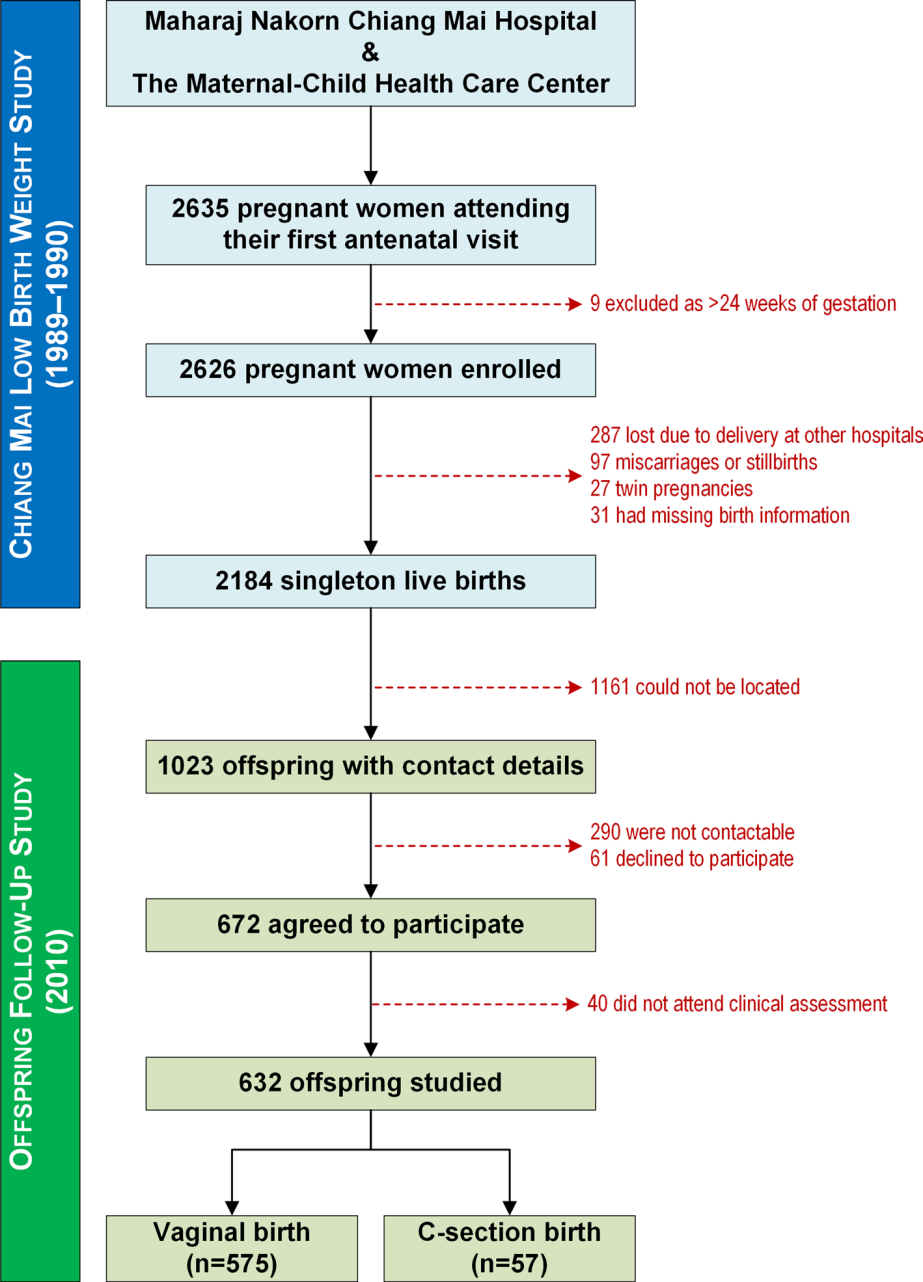
Flow diagram outlining participants' recruitment into the Chiang Mai Low Birth Weight Study (1989–1990) and subsequently to the follow-up study on the offspring (2010).

This prospective cohort study involved the offspring of mothers from the Chiang Mai Low Birth Weight Study born in 1989–1990, in Chiang Mai, Northern Thailand^15^. Briefly, 2184 women were recruited in early pregnancy (≤ 24 weeks of gestation) from two health centres, Maharaj Nakorn Chiang Mai Hospital and The Maternal-Child Health Care Center. At the time, these were the only public hospitals providing antenatal care in Chiang Mai Province^15^. In 2010, their offspring were recruited for a follow-up study at approximately 20 years of age (Fig. 1)^16^.

Demographic and clinical data were extracted from the original study database. Demographic data of interest included maternal age, maternal and paternal education levels, family income, and maternal smoking and alcohol consumption during pregnancy. Clinical data included maternal anthropometry, type of delivery, and the occurrence of pregnancy-induced hypertension, as well as the offspring's weight and gestational age at birth. Birth weights were converted into *z*-scores as per INTERGROWTH-21st standards^17^. The current education and smoking status of the offspring were obtained using questionnaires.

Follow-up participants underwent a clinical examination at the Research Institute for Health Science (RIHES), at Chiang Mai University, after an overnight fast. Participants underwent anthropometric assessments while barefoot and wearing light clothing; height was measured on a wall-mounted stadiometer to the nearest 1 mm, and weight using calibrated electronic scales to the nearest 100 g, with both measurements carried out twice to maximise accuracy. Their body mass index (BMI) was subsequently calculated, and their BMI status classified as per World Health Organization standards: underweight/normal weight < 25 kg/m^2^; overweight ≥ 25 and < 30 kg/m^2^; and obesity ≥ 30 kg/m^2^. Fasting venous blood samples were drawn to measure glucose, insulin, and lipid profile. The homeostatic model assessment of insulin resistance (HOMA-IR)^18^ was used to estimate insulin sensitivity.

Blood pressure (BP) was measured at rest in a sitting position using a digital sphygmomanometer on the left arm (Terumo ES-P311; Terumo Corporation, Tokyo, Japan). Two measurements were taken approximately 5 min apart, and their average value was recorded. Mean arterial pressure (MAP) was calculated from systolic BP (SBP) and diastolic BP (DBP) as follows:

$$MAP = DBP + \frac{1}{3}\left( {SPB - DBP} \right)$$

Types of abnormal blood pressure were defined as: systolic pre-hypertension, SBP ≥ 130 but < 140 mmHg; systolic hypertension, SBP ≥ 140 mmHg; diastolic pre-hypertension, DBP ≥ 85 but < 90 mmHg; diastolic hypertension, DBP ≥ 90 mmHg^19^. Abnormal SBP and DBP were defined as BP ≥ 130 mmHg and ≥ 85 mmHg, respectively. Maternal pregnancy-induced hypertension was defined as SBP ≥ 140 mmHg and/or DBP ≥ 90 mmHg developed after 20 weeks of gestation without proteinuria, in a previously normotensive woman^20^.

Carotid intima-media thickness was measured as a marker of atherosclerosis on the right common carotid artery, using a Philips iE33 ultrasound (Philips Medical Systems, Bothell, WA, USA) and L10-4 MHz linear array transducer.

### Statistical analyses

Demographic, familial, and birth characteristics were compared between the CS and vaginal delivery groups using Fisher's exact tests or one-way ANOVA, as appropriate. Continuous outcomes were initially compared between groups using one-way ANOVAs. Subsequently, these outcomes were compared using general linear regression models adjusting for important confounders: sex and gestational age at birth^21–23^. Additional confounders were included in these models depending on the outcome of interest: for offspring height—maternal height was also included; for offspring weight—maternal BMI^24,25^ and offspring height; for offspring BMI—maternal BMI^24,25^; and for offspring BP—mother's pregnancy-induced hypertension (yes vs no)^26^.

The prevalence of obesity, overweight/obesity, and types of abnormal BP were compared between groups using Fisher's exact tests. The likelihood of BP abnormalities was assessed using unadjusted generalised linear models and reported as relative risks. Adjusted relative risks were subsequently estimated using generalised linear models adjusting for the appropriate confounders described previously (i.e., sex, gestational age at delivery, and mother's pregnancy-induced hypertension).

There are well-described sexual dimorphisms in association with early life events, with contrasting effects on long-term health and disease observed in males and females^27^. Therefore, the interaction between delivery mode and offspring sex was also examined for all models.

Analyses were performed with SPSS v25 (IBM Corp, Armonk, NY, USA) and SAS v9.4 (SAS Institute, Cary, NC, USA). All statistical tests were two-tailed, and the significance level was maintained at 5% without adjustments for multiple comparisons, as per Rothman (1990)^28^.

### Ethics approval

The Human Experimentation Committee at the Research Institute for Health Sciences (RIHES) at Chiang Mai University provided ethical approval for this study (#17/52). All participants (i.e., mothers and offspring) provided verbal and written informed consent. This study was performed following all appropriate institutional and international guidelines and regulations for medical research, in line with the Declaration of Helsinki principles.

## Results

From the 2184 participants in the original study, 1552 were lost to follow-up, so 632 young adults were recruited into the follow-up study at a mean age of 20.6 years (Fig. 1). Our study participants were largely similar to the remainder of the original cohort (Table 1). However, a greater proportion of females was recaptured, and our participants were slightly lighter at birth (− 0.14 *z*-score), were born to mothers one year older on average, and had a median family income 14% lower (Table 1).

**Table 1:** Comparisons of demographic and birth characteristics between our study population and the original study participants who were lost to follow-up.

Characteristic	Levels	Lost to follow-up	Follow-up study	*P* value
n		1552	632	
Sex	Males	876 (56.4%)	292 (46.2%)	**<** **0.001**
	Females	676 (43.6%)	340 (53.8%)	
Birth weight *z*-score		− 0.32 ± 0.92	− 0.46 ± 0.92	**0.001**
Gestational age (weeks)		38.8 ± 2.0	39.0 ± 1.7	**0.022**
Delivery by caesarean section		57 (9.0%)	176 (11.3%)	0.13
Maternal age at childbirth (years)		25.3 ± 4.6	26.3 ± 4.6	**<** **0.001**
Maternal BMI (kg/m^2^)^a^		21.51 ± 2.86	21.34 ± 2.51	0.19
Maternal PIH^b^		40 (2.6%)	25 (4.0%)	0.10
Maternal smoking at pregnancy		14 (0.9%)	5 (0.8%)	> 0.99
Maternal alcohol consumption during pregnancy		11 (0.7%)	7 (1.1%)	0.43
Maternal education^c^	Less than high school	1127 (89.2%)	491 (90.3%)	0.56
	High school or greater	136 (10.8%)	53 (9.7%)	
Paternal education	Less than high school	1036 (82.1%)	439 (80.7%)	0.51
	High school or greater	226 (17.9%)	105 (19.3%)	
Family income (baht per month)^d^		2918 [1800, 4500]	2500 [1563, 4200]	**0.005**

Continuous data are means ± SD or median [quartile 1, quartile 3], as appropriate; categorical data are n (%).

*BMI* body mass index, *PIH* pregnancy-induced hypertension.

*P* values that are statistically significant (at *p* < 0.05) are shown in bold.

^a^BMI recorded at the first antenatal visit in the original study in 1989–1990.

^b^PIH was defined as a systolic blood pressure ≥ 140 mmHg and/or diastolic blood pressure ≥ 90 mmHg developed after 20 weeks of gestation without proteinuria, in a previously normotensive woman.

^c^There were missing data on the highest levels of education, so that the available sample sizes for the Follow-up and Lost to follow-up groups were 544 (86.1%) and 1263 (81.4%) for maternal education, respectively, and 544 (86.1%) and 1262 (81.3%) for paternal education.

^d^Income recorded at the time of maternal recruitment to the original study in 1989–1990 (i.e., not adjusted for inflation); the available sample sizes were 542 (85.8%) for the follow-up group and 1230 (79.3%) for those lost to follow-up.

Our study population included 575 (91%) individuals born by vaginal delivery and 57 (9.0%) by CS; the latter included 36 elective and 21 non-elective cases (18 cases with prolonged labour with obstruction and 3 cases with pregnancy-induced hypertension). Demographic, birth, familial, and lifestyle characteristics were similar in the two groups (Table 2), except for a higher proportion of males born by CS than vaginally (61% vs 45%; *p* = 0.018). All individuals were Thai, and no offspring had been previously diagnosed with hypertension or had been on antihypertensive medication.

**Table 2:** Demographic and birth characteristics of the study population according to the mode of delivery.

	Levels	Vaginal birth	Caesarean section	*P* value
n		575	57	
Age (years)		20.6 ± 0.5	20.5 ± 0.5	0.82
Sex	Males	256 (44.5%)	35 (61.4%)	**0.018**
	Females	319 (55.5%)	22 (38.6%)	
Birth weight (g)		2983 ± 425	2939 ± 513	0.46
Birth weight *z*-score		− 0.52 ± 0.91	− 0.67 ± 1.10	0.27
Gestational age (weeks)		39.2 ± 1.6	39.3 ± 2.2	0.70
Maternal age at childbirth (years)		26.2 ± 4.6	26.9 ± 4.5	0.28
Maternal BMI (kg/m^2^)^a^		21.31 ± 2.47	21.65 ± 2.96	0.33
Maternal PIH^b^		22 (3.8%)	3 (5.3%)	0.49
Maternal smoking at pregnancy		4 (0.7%)	1 (1.8%)	0.38
Maternal alcohol consumption during pregnancy		6 (1.0%)	1 (1.8%)	0.49
Maternal education^c^	Less than high school	405 (81.5%)	37 (78.7%)	0.79
	High school or greater	92 (18.5%)	10 (21.3%)	
Paternal education	Less than high school	351 (70.6%)	33 (70.2%)	0.85
	High school or greater	146 (29.4%)	14 (29.8%)	
Offspring education	High school or lesser	81 (18.2%)	8 (17.8%)	> 0.99
	University	363 (81.8%)	37 (82.2%)	
Family income (baht per month)^d^		2500 [1500, 4000]	3200 [1800, 5000]	0.10
Offspring current smoking		63 (10.9%)	10 (17.5%)	0.31

Continuous data are means ± SD or median [quartile 1, quartile 3], as appropriate; categorical data are n (%).

*BMI* body mass index, *PIH* pregnancy-induced hypertension.

*P* values that are statistically significant (at *p* < 0.05) are shown in bold.

^a^BMI recorded at the first antenatal visit in the original study in 1989–1990.

^b^PIH was defined as a systolic blood pressure ≥ 140 mmHg and/or diastolic blood pressure ≥ 90 mmHg developed after 20 weeks of gestation without proteinuria, in a previously normotensive woman.

^c^There were missing data on the highest level of education, so that the available sample sizes for the vaginal birth and caesarean section groups, respectively, were: maternal education [497 (86.4%) and 47 (82.5%)], paternal education [49 (86.4%) and 47 (82.5%)], and offspring education [444 (77.2%) and 45 (78.9%)].

^d^Income recorded at the time of maternal recruitment to the original study in 1989–1990 (i.e., not adjusted for inflation); the available sample sizes were 495 (86.1%) for the vaginal birth group and 47 (82.5%) for the caesarean section group.

The prevalence of obesity in the CS group was 7.0% compared to 5.2% in young adults born by vaginal delivery (*p* = 0.54; Table 3). Adjusted models did not show an increased risk of obesity [aRR 1.26 (0.46, 3.42); *p* = 0.65] or overweight and obesity [aRR 1.15 (0.69, 1.91); *p* = 0.60] in the CS compared to the vaginal delivery group, respectively.

**Table 3:** Anthropometric and cardiometabolic outcomes in a cohort of young adults in Chiang Mai (Thailand) according to delivery mode.

Outcomes	Unadjusted			Adjusted		
	Vaginal birth	C-section	*P* value	Vaginal birth	C-section	*P* value
**Anthropometry**						
Height (cm)	163.7 (163.0, 164.4)	164.8 (162.7, 167.0)	0.32	164.3 (163.8, 164.7)	163.9 (162.5, 165.3)	0.59
Weight (kg)	57.3 (56.2, 58.5)	59.5 (55.9, 63.2)	0.25	57.9 (56.9, 59.0)	57.9 (54.6, 61.1)	0.97
BMI (kg/m2)^a^	21.27 (20.91, 21.62)	21.79 (20.67, 22.91)	0.38	21.33 (20.99, 21.67)	21.54 (20.46, 22.62)	0.71
Underweight/normal weight	479 (83.9%)	45 (79.0%)	0.57	–	–	
Overweight	62 (10.9%)	8 (14.0%)		–	–	
Obesity	30 (5.2%)	4 (7.0%)		–	–	
**Blood pressure**^b^						
Systolic (mmHg)	114.1 (113.0, 115.1)	120.3 (117.1, 123.6)	**<** **0.001**	115.9 (113.8, 118.1)	120.0 (116.7, 123.4)	**0.006**
Diastolic (mmHg)	73.5 (72.7, 74.4)	76.7 (74.0, 79.4)	**0.029**	75.5 (73.5, 77.5)	77.9 (74.7, 81.1)	0.09
Mean arterial pressure (mmHg)	87.1 (86.3, 87.9)	91.2 (88.6, 93.8)	**0.003**	89.0 (87.1, 90.9)	91.9 (89.0, 94.8)	**0.024**
**Atherosclerosis marker**						
CIMT (mm)	0.439 (0.436, 0.442)	0.443 (0.435, 0.451)	0.38	0.439 (0.436, 0.442)	0.442 (0.434, 0.450)	0.47
**Glucose homeostasis**						
Fasting glucose (mg/dL)	82.7 (82.0, 83.4)	82.8 (80.5, 85.1)	0.97	82.8 (82.1, 83.5)	82.6 (80.3, 84.9)	0.86
Fasting insulin (mIU/L)	7.36 (6.96, 7.78)	7.31 (6.12, 8.73)	0.95	7.29 (6.90, 7.71)	7.46 (6.25, 8.91)	0.80
HOMA-IR	1.51 (1.42, 1.60)	1.51 (1.25, 1.81)	0.99	1.49 (1.41, 1.58)	1.53 (1.27, 1.84)	0.80
**Lipid profile**						
Total cholesterol (mg/dL)	169 (166, 171)	170 (161, 179)	0.70	168 (166, 171)	171 (161, 180)	0.66
HDL (mg/dL)	56 (55, 57)	57 (53, 61)	0.65	56 (55, 57)	58 (54, 61)	0.35
LDL (mg/dL)	96 (93, 98)	96 (88, 104)	0.88	96 (93, 98)	96 (88, 104)	0.88
Triglycerides (mg/dL)	75 (72, 78)	81 (71, 93)	0.27	76 (73, 79)	80 (70, 91)	0.49

Underweight/normal weight was defined as BMI < 25 kg/m^2^, overweight as BMI ≥ 25 but < 30 kg/m^2^, and obesity as BMI ≥ 30 kg/m^2^.

BMI status data are n (%); all other data are means, and the respective 95% confidence intervals (CI). Adjusted means are the least-squares means and respective 95% CI, adjusted for sex and gestational age at delivery, as well as: maternal height for offspring height; maternal BMI and offspring height for offspring weight; maternal BMI for offspring BMI; and mother's pregnancy-induced hypertension (yes vs no) for offspring BP.

*BMI* body mass index, *CIMT* carotid intima-media thickness, *C-section* caesarean section, *HDL* high-density lipoprotein cholesterol, *HOMA-IR* homeostatic model assessment of insulin resistance, *LDL* low-density lipoprotein cholesterol, *RR* relative risk.

*P* values that are statistically significant (at *p* < 0.05) are shown in bold.

^a^BMI data were not available for four participants born vaginally.

^b^Systolic and diastolic BP data were missing for 12 and 6 participants born vaginally, respectively, and for one participant born by C-section.

However, there were marked differences in BP between the two groups (Table 3). Young adults born by CS had SBP 6.2 mmHg higher (*p* < 0.001), DBP 3.2 mmHg higher (*p* = 0.029), and MAP 4.1 mmHg higher (*p* = 0.003) than those born vaginally (Table 3). Adjustment for covariates attenuated these differences, particularly for DBP, which was no longer different between groups (Table 3). Nonetheless, SBP and MAP remained 4.1 mmHg (*p* = 0.006) and 2.9 mmHg (*p* = 0.024) higher, respectively, among young adults born by CS (Table 3).

Compared to young adults born vaginally, the CS group had markedly higher rates of systolic pre-hypertension and systolic hypertension (Table 4). Thus, the prevalence of abnormal BP in the CS group was 2.5 times that of those born vaginally (25.0% vs 10.3%; *p* = 0.003), with an adjusted relative risk of 1.85 (95% CI 1.15, 2.98; *p* = 0.011) (Table 4). Rates of diastolic BP abnormalities were not different between groups (Table 4). There was no interaction between group and sex, indicating no sex-specific associations between CS birth and blood pressure (data not shown).

**Table 4:** Rates of blood pressure abnormalities in a cohort of young adults in Chiang Mai (Thailand) according to delivery mode.

BP type	Outcome	Vaginal birth	C-section	*P* value
Systolic BP	n	563	56	
	Normotension	505 (89.7%)	42 (75.0%)	**0.005**
	Pre-hypertension	39 (6.9%)	10 (17.9%)	
	Hypertension	19 (3.4%)	4 (7.1%)	
	RR abnormal BP	Reference	2.43 (1.45, 4.06)	**<** **0.001**
	aRR abnormal BP	Reference	1.85 (1.15, 2.98)	**0.011**
Diastolic BP	n	569	56	
	Normotension	486 (85.4%)	47 (83.9%)	0.75
	Pre-hypertension	53 (9.3%)	5 (8.9%)	
	Hypertension	30 (5.3%)	4 (7.1%)	
	RR abnormal BP	Reference	1.10 (0.59, 2.07)	0.76
	aRR abnormal BP	Reference	0.95 (0.51, 1.78)	0.88

Systolic pre-hypertension, SBP ≥ 130 but < 140 mmHg; systolic hypertension, SBP ≥ 140 mmHg; abnormal systolic BP, SBP ≥ 130 mmHg; diastolic pre-hypertension, DBP ≥ 85 but < 90 mmHg; diastolic hypertension, DBP ≥ 90 mmHg; and abnormal diastolic BP, DBP ≥ 85 mmHg.

Rates data are n (%); RR data are relative risks and the respective 95% confidence intervals (CI); aRR data are adjusted RR and respective 95% CI, adjusted for sex, gestational age at delivery, and mother's pregnancy-induced hypertension (yes vs no).

Systolic and diastolic BP data were missing for 12 and 6 participants born vaginally, respectively, and for one participant born by C-section.

*aRR* adjusted relative risk, *BP* blood pressure, *C-section* caesarean section, *RR* relative risk.

*P* values that are statistically significant (at *p* < 0.05) are shown in bold.

There were no differences in anthropometry, glucose metabolism, lipid profile, or carotid intima-media thickness between groups (Table 3).

## Discussion

This prospective cohort study found that young adults born by CS had higher blood pressure than peers born vaginally, with nearly twice the likelihood of elevated SBP. Overall, few studies have examined associations between CS and cardiovascular risk factors in the offspring, and the results have been conflicting. Consistent with our findings, in a Brazilian birth cohort, young adults born by CS were 1.5 times more likely to have hypertension than those born vaginally; however, the authors did not report a difference in mean SBP or DBP by birth mode^29^. In another Brazilian birth cohort study, SBP was 1.4 mmHg higher, and DBP was 1.1 mmHg higher for male (but not female) young adults born by CS^3^. Among Chinese children aged 4–7 years, those born by elective CS were more likely to have a BP > 90th percentile for age and sex, and had SBP 2.9 mmHg higher than children born vaginally^6^. In contrast, however, a study in Dutch children and one in Danish young adults did not observe an association between CS delivery and blood pressure^4,30^.

In our cohort, CS delivery was not associated with the risk of overweight or obesity. The study in Chinese children reported a small but significant increase in mean BMI in children born after elective CS^6^, while a study in Vietnam observed an increased risk of obesity in 8-year-olds born by CS^31^. Two meta-analyses have reported greater odds of overweight and obesity after CS delivery^32,33^. Interestingly, among 3-year-olds in Ireland, the risk of obesity was 56% greater in those born by emergency CS but not elective CS^7^. In contrast, the opposite was reported among Singaporean 1-year-olds, where an increased risk of overweight was observed among those born by elective CS but not by emergency CS^34^. There are, however, conflicting findings, and another study in Ireland found no association between delivery mode and risk of overweight in the first five years of life^35^. The lack of consistency between studies may be explained in part by differences in both the prevalence and indications for CS between different medical systems, as well as sociodemographic differences in study participants^2^. Only 7% of children in the Dutch cohort were born by CS^30^, while CS births made up more than a quarter of those in the Irish cohorts^7,35^. Besides, CS infants make up a small proportion of participants in many studies and may be diverse in terms of their medical histories leading to CS. Notably, a large study in the US on 16,140 siblings reported that within families, CS delivery was not associated with a higher BMI z-score at 5 years of age^36^. The authors, therefore, suggested that unmeasured confounders (such as maternal BMI and sociocultural factors) likely accounted for the reported associations between CS delivery and increased BMI in many studies^36^.

Though there is some evidence that exposure to microflora during vaginal birth may lead to more optimal gut microbiome development in infants, this proposition has been rejected by some authors^37^. Epigenetics has also been suggested to play a role in the relationship between CS and cardiovascular outcomes. Methylation of genes related to the regulation of food responses, glycolysis, and ketone metabolic processes have been reported to be higher for CS-born infants across the first few days of life, but the consequences of these changes for gene expression and future health risks are yet to be understood^38^. Further, the relationship between CS and offspring blood pressure is influenced by other maternal factors; in particular, women with either pre-existing or pregnancy-specific hypertension are more likely to undergo CS and also more likely to have offspring with higher blood pressure across childhood and adolescence^39^. Of note, our blood pressure analyses adjusted for the mother's pregnancy-induced hypertension, suggesting that other factors beyond maternal blood pressure are at play.

Our study's main limitation was the relatively low participation rate (29%) from the original birth cohort at the 20-year follow-up, which might have resulted in some selection bias. For example, at recruitment for the original study, median income among our follow-up participants was 14% lower than those who were lost. However, importantly, parents in both groups had similar education levels, with other demographic characteristics also largely similar. While we recorded information on the offspring's education, their socioeconomic status was not formally assessed and therefore not accounted for in our statistical analyses, but data on their mothers indicated that the CS and vaginal birth groups had similar demographic and lifestyle characteristics. Furthermore, data on dietary habits that can affect blood pressure (such as salt intake and alcohol consumption) were not recorded. Also, while our sample size was still relatively large (n = 632), there was a low rate of CS in our cohort (9.0%), which likely limited our statistical power to detect potential differences between groups, particularly regarding CS indication (i.e., elective vs emergency). However, the CS rate in our follow-up participants and the original cohort (10.6%) simply reflected general CS rates in Chiang Mai (and Thailand) at the time. In 1992, 11.3% of births occurred by CS at the Maharaj Nakorn Chiang Mai Hospital^40^, with national CS rates of 15.2% across Thailand^41^. Nonetheless, our study's major strength is that, to our knowledge, this is the first investigation to examine the associations between CS delivery and long-term health in the offspring in Thailand.

In conclusion, our study showed that CS delivery was associated with an increased risk of blood pressure in young adult offspring in Thailand. With the increasing rates of CS delivery in this country^1^, further studies are needed to clarify whether these associations persist in the long-term and the potential underlying mechanisms. Importantly, it is still unclear whether the observed increase in blood pressure at the age of 20 years will lead to greater cardiovascular morbidity and mortality.

## Data Availability

The anonymised data on which this manuscript was based could be made available to other investigators upon bona fide request, and following all the necessary approvals (including ethics) of the detailed study proposal and statistical analyses plan. Any queries should be directed to Prof Kittipan Rerkasem (rerkase@gmail.com).
